# Effectiveness of second generation antipsychotics: A systematic review of randomized trials

**DOI:** 10.1186/1471-244X-8-31

**Published:** 2008-04-25

**Authors:** Erik Johnsen, Hugo A Jørgensen

**Affiliations:** 1 Haukeland University Hospital, Division of Psychiatry, Sandviken, P.O.Box 23, N-5812, Bergen, Norway; 2 Department of Clinical Medicine, Section Psychiatry, University of Bergen, N-5020 Bergen, Norway

## Abstract

**Background:**

Systematic reviews based on efficacy trials are inconclusive about which second generation antipsychotic drug (SGA) should be preferred in normal clinical practice, and studies with longer duration and more pragmatic designs are called for. Effectiveness studies, also known as naturalistic, pragmatic, practical or real life studies, adhere to these principles as they aim to mimic daily clinical practice and have longer follow-up.

**Objective:**

To review the head-to-head effectiveness of SGAs in the domains of global outcomes, symptoms of disease, and tolerability.

**Methods:**

Searches were made in Embase, PubMED, and the Cochrane central register of controlled trials for effectiveness studies published from 1980 to 2008, week 1. Different combinations of the keywords antipsychotic*, neuroleptic* AND open, pragmatic, practical, naturalistic, real life, effectiveness, side effect*, unwanted effect*, tolera* AND compar* AND random* were used.

**Results:**

Sixteen different reports of randomized head-to-head comparisons of SGA effectiveness were located. There were differences regarding sample sizes, inclusion criteria and follow-up periods, as well as sources of financial sponsorship. In acute-phase and first-episode patients no differences between the SGAs were disclosed regarding alleviating symptoms of disease. Olanzapine was associated with more weight gain and adverse effects on serum lipids. In the chronic phase patients olanzapine groups had longer time to discontinuation of treatment and better treatment adherence compared to other SGAs. The majority of studies found no differences between the SGAs in alleviating symptoms of psychosis in chronically ill patients. Olanzapine was associated with more metabolic adverse effects compared to the others SGAs. There were surprisingly few between-drug differences regarding side effects. First generation antipsychotics were associated with lower total mental health care costs in 2 of 3 studies on chronically ill patients, but were also associated with more extrapyramidal side effects compared to the SGAs in several studies.

**Conclusion:**

In chronically ill patients olanzapine may have an advantage over other SGAs regarding longer time to treatment discontinuation and better drug adherence, but the drug is also associated with more metabolic side effects. More effectiveness studies on first-episode psychosis are needed.

## Background

Results from effectiveness trials on antipsychotics have been awaited with anticipation. Several ongoing or recently completed effectiveness studies in both the USA and Europe have been expected to supplement the base of evidence regarding the clinical use of antipsychotic drugs. According to present international recommendations most second generation antipsychotics (SGAs) other than clozapine are considered first line drugs for a patient suffering from psychosis [1-4]. Despite differing chemical and pharmacological properties double-blind, randomized, controlled clinical trials (RCTs) fail to consistently demonstrate superiority for any of these drugs on efficacy measures. This is reflected in the inconclusiveness of systematic reviews on antipsychotics which call for longer-term trials with more pragmatic designs [5-12]. RCTs of efficacy are indeed important for new candidate antipsychotics in establishing superiority over placebo and/or non-inferiority compared to reference antipsychotics. Several methodological issues concerning sample selection and the rigid experimental environment could restrict the generalizability of the efficacy trial results, however. Selection bias for one is a major concern. The proportion actually included in the studies are in many instances difficult to quantify, as the number of patients initially assessed for eligibility rarely is disclosed in the scientific papers. Where reported, however, this proportion in different studies is found to be as low as 7–27% [13-16]. Then the results from efficacy trials are extrapolated to clinical populations that may have different characteristics. Adding to this, patients in normal clinical practice commonly use more than one psychotropic drug [17-20]. As to what extent these combinations will modulate the antipsychotic drug effects and tolerability outcomes registered in RCTs of efficacy remains to be answered.

The trials of effectiveness have been launched in recent years to address some of the limitations of efficacy trials. Effectiveness trials, as opposed to efficacy trials, take a more pragmatic approach, and could be a rational approach to the problems related to selection and experimental settings. The trial design is also frequently labelled "naturalistic", "real-life", "pragmatic", or "practical". These terms are not strictly defined, but the common denominator is that both sample and experimental environment should resemble daily clinical practice. The core question of effectiveness trials is how a treatment works under normal clinical circumstances, not in the ideal situations in the efficacy setting [21]. Another important feature of effectiveness trials is that outcome measures also include more global aspects of patient functioning, such as quality of life measures. Nasrallah et al. [22] propose a model where effectiveness is measured according to four domains: Symptoms of disease, treatment burden, disease burden, and health and wellness. Using a modified, 3-domain version of the effectiveness definition proposed by Nasrallah et al., the aims of the review were to investigate whether effectiveness trials have disclosed differences between SGAs in the domains of global outcomes, symptoms of disease, and how between-drug differences in adverse effect profiles are expressed in naturalistic settings.

## Objective

To review the head-to-head effectiveness of SGAs in the domains of global outcomes, symptoms of disease, and tolerability.

## Methods

### Types of studies

All relevant original randomized, controlled clinical effectiveness trials with head-to-head comparisons of second generation antipsychotics were eligible for inclusion. Trials were categorized as effectiveness studies if there was a statement from the authors that a naturalistic, pragmatic, practical, or real life study design was used, or if the methodology section was presented in corresponding terms.

Studies that restricted the use of concomitant medications were excluded. In every day clinical practice, adjunctive psychoactive medications such as antidepressants and mood stabilizers are commonly used concomitantly with antipsychotics. This practice is in many instances in accordance with treatment guidelines [1,23]. Exclusion from studies of patients that qualify for adjunctive antidepressants or mood stabilizers, or restricting them from using these drugs, are in the opinion of the authors in conflict with the pragmatic principle of effectiveness studies. Only restrictions on the use of more than one antipsychotic drug were tolerated. Conference abstracts were excluded. Clozapine trials were excluded as this agent is commonly not regarded a first-line treatment option.

### Types of participants

Adult patients (over 16 years of age) with a diagnosis of schizophrenia or schizophrenia like disorder such as delusional disorder, schizoaffective disorder, schizophreniform psychosis.

### Types of intervention

1. First line second generation antipsychotic drugs: aripiprazole, olanzapine, quetiapine, risperidone, ziprasidone.

2. First generation antipsychotic drugs: chlorpromazine, haloperidole, perphenazine (when included as comparators in head-to-head comparisons of first line second generation antipsychotic drug).

3. Placebo (when included as a comparator group in head-to-head comparisons of first line second generation antipsychotic drug).

### Types of outcome measures

#### 1. Global outcomes

1.1 Time until discontinuation of assigned antipsychotic drug for any and specific causes.

1.2 Compliance with assigned antipsychotic drug.

1.3 Duration of hospitalisation.

1.4 Total mental health treatment costs.

1.5 Quality of life – as defined by each of the studies.

#### 2. Symptoms of disease

2.1 Average score/change in mental state – as defined by each of the studies.

#### 3. Tolerability

3.1 Extrapyramidal side effects.

3.2 Metabolic side effects.

3.3 Prolactin related symptoms.

3.4 Other adverse effects, general and specific.

#### 4. Concomitant medication

4.1 Incidence of antidepressants, antiparkinson drugs, mood stabilizers, sedatives.

### Search strategy for identification of studies

#### 1. Electronic searching

Searches were made in Embase, PubMED and the Cochrane central register of controlled trials for articles published from 1980 to 2008, week 1 using the phrase:

[(unwanted effect* AND compar* AND antipsychotic* AND random*) OR (unwanted effect* AND compar* AND neuroleptic* AND random*) OR (side effect* AND compar* AND neuroleptic* AND random*) OR (side effect* AND compar* AND antipsychotic* AND random*) OR (tolera* AND compar* AND neuroleptic* AND random*) OR (tolera* AND compar* AND antipsychotic* AND random*) OR (efficacy AND compar* AND neuroleptic* AND random*) OR (efficacy AND compar* AND antipsychotic* AND random*) OR (pragmatic AND antipsychotic* AND random*) OR (pragmatic AND neuroleptic* AND random*) OR (real life AND neuroleptic* AND random*) OR (real life AND antipsychotic* AND random*) OR (naturalistic AND antipsychotic* AND random*) OR (naturalistic AND neuroleptic* AND random*) OR (open AND neuroleptic* AND random*) OR (open AND antipsychotic* AND random*) NOT (antipsychotic* AND tourette*) NOT (neuroleptic* AND tourette*) NOT (neuroleptic* AND mania) NOT (antipsychotic* AND mania)].

#### 2. Reference searching

The references of all identified studies were inspected for more trials.

### Methods of the review

#### 1. Selection of trials

All citations were inspected by the principal reviewer (EJ). Studies were selected on the basis of abstracts, and in cases of doubt, the full text articles were consulted. If doubt remained, this was resolved by discussion with HAJ.

#### 2. Data collection

Data were extracted from both text and tables of the original papers.

#### 3. Data synthesis

##### 3.1 Data presentation

Data on effectiveness was evaluated and grouped according to the three domains: global outcomes, symptoms of disease, and tolerability.

Outcomes were compared between the treatment groups and graded according to statistically significant inferiority (<), statistically significant superiority (>), or equality (=) between groups. Level of significance was set at 0.05.

##### 3.2 Sensitivity analyses

###### 3.2.1 First episode

Results from those experiencing their first episode of psychosis are analyzed separately.

###### 3.2.2 Intention to treat analyses

When result gained from both intention to treat analyses (ITT) and observed cases (OC) are available, both are included. P-values are only given for ITT analyses if the results of both ITT and OC analyses are statistically significant.

#### 4. Heterogeneity

Results from different trials are analyzed separately because of study heterogeneity.

#### 5. Addressing funding bias

The funding parties of the studies were registered, as the matter of conflict of interest in research has become a growing concern in recent years.

## Results

### Description of studies

The search provided more than a thousand different hits in the databases, of which the vast majority were non-relevant, animal studies or studies on efficacy. A total of 16 different reports from 10 randomized trials of effectiveness with head-to-head comparisons of SGAs were located. The methodological aspects of the trials are presented in Table 1. Five studies present data from different phases of the Clinical Antipsychotic Trials of Intervention Effectiveness (CATIE) [31,35-38]. The studies by Ritchie et al. [33,34] include elderly patients exclusively, as opposed to the others. The study sample is the same in the two studies by Ritchie et al., but the studies present data from different phases of the same trial. There are differences across all studies concerning source of sample recruitment (in- or outpatients), phase of illness (first episode or chronic), antipsychotic drug doses, and level of diagnostic specificity in the inclusion criteria. The study by McEvoy et al. [25] and the CATIE studies applied double-blinding, the studies by Robinson et al. [26] and McCue et al. [29] had rater-blinding, whereas the rest were without blinding.

**Table 1:** Methodology

**Study**	**Comparators**	**Doses (DDD)**	**Diagnoses**	**Age**	**Phase/status**	**N_0_/N_itt_/N_1_**	**Follow-up**	**Funding**
**FIRST EPISODE**								
								
Crespo-Facorro et al. [24]	Haloperidol Olanzapine Risperidone	5.4 mg (0.68) 15.3 mg (1.53) 4.0 mg (0.80)	Schizophrenia and related, non-affective psychosis	15–60 years	Acute, first episode in- and outpatients	172/172/165	6 weeks	Independent
								
McEvoy et al. [25]	Olanzapine Quetiapine Risperidone	11.7 mg (1.17) 506.0 mg (1.27) 2.4 mg (0.5)	Schizophrenia, schizoaffective- schizophreniform disorder	16–40 years	Acute, first episode in- and outpatients	400/-/119	12 months	AstraZeneca company
								
Robinson et al. [26]	Olanzapine Risperidone	11.8 mg (1.18) 3.9 mg (0.78)	Schizophrenia, schizoaffective- schizophreniform disorder	16–40 years	Acute, first episode	120/112/81	4 months	Independent
								
**ACUTE PHASE**								
								
Chrzanowski et al. [27]	Aripiprazole Olanzapine	22.0 mg (1.47) 14.2 mg (1.42)	Schizophrenia	≥ 18 years	Acute psychosis/chronic stable in- and outpatients	214/214/147	52 weeks	BMS/Otsuka
								
Kraus et al. [28]	Olanzapine Risperidone	12.4 mg (1.24) 3.4 mg (0.39)	Psychosis; positive symptoms	18–60 years	Acute, inpatients	85/-/68	Not defined	Independent
								
McCue et al. [29]	Aripiprazole Haloperidol Olanzapine Quetiapine Risperidone Ziprasidone	21.8 mg^1 ^(1.45) 16.0 mg ^1^(2.00) 19.1 mg ^1^(1.91) 652.5 mg^1 ^(1.63) 5.2 mg^1 ^(1.04) 151.2 mg^1 ^(1.89)	Schizophrenia, schizoaffective- schizophreniform disorder	≥ 18 years	Acute, inpatients	327/319/301	3 weeks	Independent
								
**CHRONIC PHASE**								
								
Jerrel [30]	Olanzapine Risperidone Haloperidol Haloperidol (d) Fluphenazine (d)	13.8 mg (1.38) 5.3 mg (1.06) 15.5 mg (1.93) 6.3 mg (1.92) 3.0 mg (0.43)	Schizophrenia and schizoaffective disorder	18–54 years	Chronic, inpatients acute care	108/-/84	12 months	Independent
								
Lieberman et al. [31]	Olanzapine Perphenazine Quetiapine Risperidone Ziprasidone	20.1 mg (2.01) 20.8 mg (0.69) 543.4 mg (1.36) 3.9 mg (0.78) 112.8 mg (1.41)	Schizophrenia	18–65 years	Chronic, not treatment resistant, in- and outpatients	1493/1432/371	18 months	Independent
								
Mullen et al. [32]	Quetiapine Risperidone	329.0 mg (0.82) 5.0 mg (1.00)	Psychotic disorders^2^	> 18 years	Outpatients	728/-/493	4 months	AstraZeneca company
								
Ritchie et al. [33]	Olanzapine Risperidone	9.9 mg (0.99) 1.7 mg (0.34)	Schizophrenia	> 60 years	Outpatients, switch from FGAs	66/61/52	switch period (mean 40 days)	Eli Lilly company
								
Ritchie et al. [34]	Olanzapine Risperidone	12.4 mg (1.24) 2.0 mg (0.39)	Schizophrenia	> 60 years	Outpatients, switch from FGAs	66/61/42	6 months	Eli Lilly company
								
Rosenheck et al. [35]	Olanzapine Perphenazine Quetiapine Risperidone Ziprasidone	Not disclosed - - - -	Schizophrenia	18–65 years	Chronic, not treatment resistant, in- and outpatients	1493/1424/363	18 months	Independent
								
Stroup et al. [36]	Olanzapine Quetiapine Risperidone Ziprasidone	20.5 mg (2.05) 565.2 mg (1.41) 4.1 mg (0.82) 115.9 mg (1.45)	Schizophrenia	18–65 years	Chronic, not treatment resistant, in- and outpatients, discontinued previous SGA because of intolerability	444/333/88	18 months	Independent
								
Stroup et al. [37]	Olanzapine Quetiapine Risperidone	20.7 mg (2.07) 586.1 mg (1.47) 3.7 mg (0.74)	Schizophrenia	18–65 years	Chronic, not treatment resistant, in- and outpatients, discontinued Perphenazine	115/114/37	18 months	Independent
								
Swartz et al. [38]	Olanzapine Perphenazine Quetiapine Risperidone Ziprasidone	Not disclosed - - - -	Schizophrenia	18–65 years	Chronic, not treatment resistant, in- and outpatients	1493/985/455	18 months	Independent
								
Tunis et al. [39]	Olanzapine Risperidone Haloperidol Perphenazine	13.5 mg (1.35) 4.9 mg (0.99) 11.0 mg (1.37) 14.2 mg (0.47)	Schizophrenia, schizoaffective- schizophreniform disorder, ≥ 18 on the BPRS	≥ 18 years	Primarily outpatients	664/-/455	12 months	Eli Lilly company

**Abbreviations: **Comparators = Antipsychotics being compared in the studies; Doses = Mean daily doses of the antipsychotics; DDD = Defined Daily Doses of the antipsychotics; Diagnoses = Diagnoses defined in inclusion criteria; Age = Age of study participants; Phase = Phase of psychotic illness of study participants: acute or chronic; Status = Status of study participants: first episode, inpatients, outpatients; N_0 _= Number of participants at baseline; N_itt _= Number in intention to treat group (not disclosed in all studies as indicated by (-)), N_1 _= Number of completers; Follow-up = Duration of study follow-up; Funding = Financial support of study; BMS = Bristol-Myers Squibb; Otsuka = Otsuka America Pharmaceuticals, inc.; Independent = Study financially supported by sources independent of the pharmaceutical industry. A = Aripiprazole, FGAs = First generation antipsychotics, H = Haloperidol, O = Olanzapine, P = Perphenazine, Q = Quetiapine, R = Risperidone, Z = Ziprasidone, BPRS = the Brief Psychiatric Rating Scale, d = depot formulation.

^1 ^Mean maximum daily dosage

^2 ^Schizophrenia, schizophreniform -, schizoaffective -, delusional disorder, major depressive disorder with psychotic features, Alzheimer's dementia with psychotic symptoms, vascular dementia, dementia due to substance abuse

### Global outcomes

Table 2 gives an overview of global outcomes. The most consistent difference is that chronic patients treated with olanzapine used this antipsychotic drug for a longer time or with better adherence compared to the other SGAs [30,31,36,37,39]. Regarding total mental health treatment costs, two studies found significantly lower costs using FGAs compared to SGAs [30,35], whereas one study found the total costs to be equal across the drug generations [38]. Costs were only estimated in chronic-phase studies.

**Table 2:** Global outcomes

**Studies**	**Main results (*outcome measures*)**
**FIRST EPISODE**	
	
Crespo-Facorro et al. [24]	No global outcomes
McEvoy et al. [25]	**O = Q = R ***(time to discontinuation for any cause, for inadequate therapeutic effect, for unacceptable side effects, for patients decision*)
Robinson et al. [26]	**O = R **(*rate of discontinued intervention*)
	
**ACUTE PHASE**	
	
Chrzanowski et al. [27]	No global outcomes
Kraus et al. [28]	**O = R **(*Duration of hospitalisation*)
McCue et al. [29]	**H = O = R > A = Q = Z **(*no longer needing acute in-patient care*)(p < 0.0001) **A = H = O = Q = R = Z **(*time until treatment classified as effective, i.e. no longer needing acute in-patient care, medication ineffective as judged by discontinuation because of no significant improvement after at least 3 weeks of treatment, side-effects or significant deterioration of patient's mental state*.)
	
**CHRONIC PHASE**	
	
Jerrel [30]	**O **(p = 0.02) **& R **(p = 0.005) **> FGA **(*total mental health treatment costs*)^1^ **O > R & FGA **(*odds of compliance*) (p < 0.001) **O = R = FGA **(*psychosocial functioning (RFS), time to discharge and rehospitalisation, client satisfaction (CSQ)*).
Lieberman et al. [31]	**O > Q **(p < 0.001) **& R **(p = 0.002) (*Time to discontinuation for any cause*) **O > P **(p < 0,001) **= Q **(p < 0.001) = R (p < 0.001) (*Time to discontinuation for lack of efficacy*) **O = Q = P = R = Z **(*Time to discontinuation for intolerable side effects*) **O > Q **(p < 0.001) = **R **(p = 0.008) (*time to discontinuation due to patient's decision*) **Q > Z > P > R > O **(p < 0.001) (*hospitalisation for exacerbation of schizophrenia*)
Mullen et al. [32]	No global outcomes
Ritchie et al. [33]	**O = R **(*quality of life, except O > R for psychological well-being *(p = 0.02)
Ritchie et al. [34]	**O > R **(*physical health *(p = 0.03), *social relationships *(p = 0.015); *overall quality of life *(p = 0.04), *health satisfaction *(p = 0.03)) **O = R **(*psychological well-being, environmental domain*)
Rosenheck et al. [35]	**O = Q = R = Z > P **(*total health care costs*) (p < 0.001) **O & Q & R & Z > P **(*total medication costs*) (p < 0.0001) **O > Q & R & Z **(*drug costs*) (p = ?) **P > R **(*Quality Adjusted Life Years ratings*) (p < 0.005) **O = P = Q = R = Z **(*quality of life measures*)
Stroup et al. [36]	**O = R > Q **(p < 0.05) **= Z **(p < 0.01) (*time to discontinuation for any cause*) **O = Q = R = Z **(*time to discontinuation for lack of efficacy/for intolerable side effects*) **O > Q = Z **(*time to discontinuation for patients who previously discontinued SGA due to inefficacy*) (p < 0.01) **R > Q **(*time to discontinuation for patients who previously discontinued SGA due to inefficacy*) (p < 0.05) **Q > Z > R > O **(p = 0.02) (*hospitalisation for exacerbation of schizophrenia*)
Stroup et al. [37]	**O **(p = 0.02) = **Q **(p = 0.04) **> R **(*Time to discontinuation for all causes*) **O = Q = R **(*cause of discontinuation*)
Swartz et al. [38]	**O = P = Q = R = Z **(*change in the Quality of life total and subscores at 6, 12 and 18 months*)
Tunis et al. [39]	**O = R = FGAs **(*total costs*) **O > R & FGA **(*antipsychotic costs*) (p < 0.001) **R **(p < 0.001) > **FGA **(p < 0.001) > **O**(*rate of switching from assigned antipsychotic agent*) **O > R **(p = 0.002) **& FGA **(p = 0.043) (*mean social responder days*^2^)

**Abbreviations: **A = Aripiprazole, FGAs = First generation antipsychotics, H = Haloperidol, O = Olanzapine, P = Perphenazine, Q = Quetiapine, R = Risperidone, Z = Ziprasidone CSQ-8 = the Client Satisfaction Questionaire-8; RFS = the Role Functioning Scale

^1 ^Total mental health treatment costs: Inpatient, outpatient and psychiatric medication costs.

^2 ^Social response is defined as maintaining a high level of satisfaction with social relationships (for patients reporting a baseline score of at least 18), or by improving at least 33% of possible improvement.

? = The authors state that the difference is significant, but do not reveal the p-value for this comparison.

In first episode patients the comparators performed equally.

### Symptoms of disease

Regarding symptoms of disease (Table 3) the studies on acute-phase patients including first-episode patients disclosed no differences between comparator drugs in the clinical global impression [24,25]; overall symptoms of psychosis [24-27,29], depression or mania [24]. In one study comparing olanzapine and risperidone in first-episode patients the response seemed to be more stable in the risperidone group, as measured by the Schedule for Affective Disorders and Schizophrenia Change Version with psychosis and disorganization items (SADS-C+PD) and the Clinical Global Impression scale (CGI) [26]. In chronic patients the results were less uniform. The majority of the studies found the SGAs to be equally effective for symptoms of psychosis [30-35,37]. Olanzapine was more effective for symptoms of psychosis compared to quetiapine and ziprasidone as measured by PANSS in one study [36]. In one study quetiapine was significantly more effective for symptoms of depression compared to risperidone as measured by the Hamilton Rating Scale for Depression (HAM-D) [32], whereas olanzapine and risperidone was equally effective on this outcome measure in 3 studies [30,33,34]. No differences between the SGAs were disclosed on the CGI scale.

**Table 3:** Symptoms of disease

**Studies**	**Main results (*rating scales/outcome measures*)**
**FIRST EPISODE**	
	
Crespo-Facorro et al. [24]	**O = R = H **(*CGI-S, BPRS, SAPS, SANS, HAM-D, CDS, YMRS, response^1 ^rate, mean time to response*)
McEvoy et al. [25]	**O = Q = R **(*PANSS total change*) **O > Q **(*PANSS positive subscale reduction*) (p = 0.013) **O = Q = R **(*response rate*^2^)
Robinson et al. [26]	**O = R **(*cumulative response*^3^*rates, mean time to response*) **O = R **(*delusions, hallusinations, thought disorders (SADS-C+PD positive symptoms items); affective flattening, alogia, avolition-apathy, asociality-anhedonia (SANS)*) **R > O **(*mean length of time that subjects maintained the of responder status*) (CI= 8.6-10.4 vs. 5.6-7.7)
	
**ACUTE PHASE**	
	
Chrzanowski et al. [27]	**A = O **(*PANSS, CGI-I*)
Kraus et al. [28]	No outcomes
McCue et al. [29]	**A = H = O = Q = R = Z **(*BPRS change*)
	
**CHRONIC PHASE**	
	
Jerrel [30]	**O = R = FGA **(*PANSS, BPRS, DIS-III-R Depression and Mania symptoms*)
Lieberman et al. [31]	**O = P = Q = R = Z **(*PANSS, CGI*) **O > Q **(p < 0.001) **& R **(p = 0.002) **& P **(p = 0.013) (*duration of successful treatment^4^*) **R > Q **(*duration of successful treatment*) (p = 002)
Mullen et al. [32]	**Q = R **(*PANSS*) **Q > R **(*HAM-D*) (p = 0.028) **Q = R **(*CGI-I*)
Ritchie et al. [33]	**O = R **(*BPRS, SANS, MADRS, MMSE*)
Ritchie et al. [34]	**O = R **(*BPRS, SANS, MADRS, MMSE*)
Rosenheck et al. [35]	**O = P = Q = R = Z **(*PANSS*)
Stroup et al. [36]	**O > Q **(p = 0.005) **& Z **(p = 0.005) (*PANSS*) **O > Q **(p < 0.001) **& Z **(p < 0.001) **& R **(p < 0.02) (*PANSS positive symptoms*) **R > Z **(p < 0.03) (*PANSS positive symptoms*) **O = Q = R = Z **(*CGI*)
Stroup et al. [37]	**O = Q = R **(*PANSS*) **O = Q = R **(*CGI at endpoint*)
Swartz et al. [38]	No outcomes
Tunis et al. [39]	**O > FGA **(*mean clinical responder days^5^*) (p = 0.025)

**Abbreviations: **A = Aripiprazole, FGAs = First generation antipsychotics, O = Olanzapine, P = Perphenazine, Q = Quetiapine, R = Risperidone, Z = Ziprasidone, CGI-I = Clinical Global Improvement Scale, CGI = CGI-S = Clinical Global Impressions- Severety of Illness Scale, PANSS = the Positive And Negative Syndrome Scale, BPRS = the Brief Psychiatric Rating Scale, SAPS = the Scale for the Assessment of Positive Symptoms, SANS = the Scale for the Assessment of Negative Symptoms, DIS-III-R = Diagnostic Interview Schedule III-R Depression and Mania Modules, HAM-D = the Hamilton Rating Scale for Depression, MADRS = the Montgomery Åsberg Depression Rating Scale, MMSE = Mini-mental state examination.

^1 ^Response = 40% or greater BPRS total improvement from baseline.

^2^Response = ≤ 3 for all PANSS items and = 3 for the CGI-S.

^3 ^Response = mild or better on SADS-C+PD positive symptoms items plus CGI much or very much improved.

^4 ^Successful treatment time is defined as number of months of treatment in which patients had a CGI Scale score of at least 3 or a score of 4 with an improvement of at least two points from baseline.

^5 ^Clinical response is defined as a BPRS score less than 18.

Subanalyses on efficacy measures are omitted

[24], [25] and [27] provide analyses on both last observation carried forward (LOCF) and observed cases. The main findings are the same, but only p-values for LOCF data are displayed in the table.

### Tolerability

Concerning the tolerability outcomes (Table 4) the most consistent differences between the SGAs were more weight gain and adverse effects on serum lipids in the olanzapine treated groups. This was found in both acute phase including first-episode patients and chronically ill patients [24-27,31,36,37,39]. Only one study comparing olanzapine and risperidone in the elderly found no significant difference between the drugs with regards to weight gain [34]. Regarding sexual dysfunction and related symptoms only one study used this outcome measure in the acute phase trials and found no difference between haloperidol, olanzapine and risperidone. In the studies including chronic patients no differences between olanzapine, quetiapine, risperidone and ziprasidone were found in one study on this outcome measure despite significantly higher mean prolactin change in the risperidone group compared to the others [31], and no difference was found between olanzapine and risperidone in the elderly [34]. Risperidone was associated with more sexual dysfunctions and gynecomastia/galactorrhoea compared to olanzapine, quetiapine and ziprasidone in CATIE patients who had previously discontinued previous treatment with an SGA [36]; whereas olanzapine, quetiapine and risperidone were equally associated with sexual dysfunctions in those who had previously discontinued perphenazine [37]. In both the latter studies risperidone treated groups had more prolactin elevation than the other SGAs. The incidence of extrapyramidal symptoms (EPS) were equally distributed among the comparator SGA groups across all studies using this outcome measure, whereas the FGAs were associated with significantly more EPS or discontinuation owing to EPS in 3 [24,31,39] of 5 studies, whereas 2 studies did not find EPS differences between FGAs and SGAs [29,30]. The last study including an FGA arm did not have outcomes on this measure.

**Table 4:** Tolerability outcomes (*rating scales/outcome measures*)

**Studies**	**Main results**
**FIRST EPISODE**	
	
Crespo-Facorro et al. [24]	**O = H = R **(*AIMS*) **O = H = R **(*asthenia, tremor, increased/reduced salivation, erectile and ejaculatory dysfunction, amenorrhoea (UKU)*) **H > O & R **(*treatment emergent parkinsonism (SAS) *(p < 0.001) *and akathisia (BARS) *(p < 0.001)) **H > O > R **(*concentration difficulties *(p = 0.044), *sleepiness/sedation *(0.012) *increased duration of sleep *(p = 0.033) *(UKU)*) **H > R > O **(*rigidity *(p = 0.005), *hypokinesia *(p = 0.006), *akathisia *(p = 0.029) (*UKU*)) **O > R > H **(*weight gain*) (p < 0.001) (*UKU*)
McEvoy et al. [25]	**O = Q = R **(*SAS, BARS, AIMS*) **O > Q & R**(*weight gain and BMI change, and male weight gain and BMI change*) (p < 0.01) **O > Q **(*female weight gain and BMI change*) (p < 0.001) **R > Q **(*female weight gain and BMI change*) (p < 0.01) **Q & R < O **(*weight gain > 7 %*) (p < 0.05) **O > Q **(*male weight gain > 7 % (week 12 only)*) (p < 0.01) **O > R **(*male weight gain > 7 %*) (p < 0.05) **O > Q **(*female weight gain > 7% (week 12 only)*) (p < 0.01) **R > Q **(*female weight gain > 7 % (week 52 only)*) (p < 0.05) **O > Q **(*BMI increase ≥ 1 unit*) (p < 0.05) **O > R **(*BMI increase ≥ 1 unit (week 12 only)*) (p < 0.01) **O > Q **(*male BMI increase ≥ 1 unit (week 12 only)*) (p < 0.05) **O > R **(*male BMI increase ≥ 1 unit*) (p < 0.05) **O > Q **(*female BMI increase ≥ 1 unit (week 12 only)*) (p < 0.01) **O > R **(*female BMI increase ≥ 1 unit (week 12 only)*) (p < 0.05) **O > R **(*female waist circumference > 35 inches change (week 12 only)*) (p < 0.05) **O & Q > R **((*fasting TG levels change (week 52 only*) (p < 0.05) **Q > R **(*increase of proportion with fasting TG level > 150 mg/dl*) (p < 0.01) **Q > O **(*increase of proportion with fasting TG level > 150 mg/dl (week 12 only)*) (p < 0.05) **Q > R **(*fasting total cholesterol change (week 52 only)*) (p < 0.05) **O > Q **(*negative fasting HDL cholesterol change and male negative fasting HDL cholesterol change (week 52 only)*) (p < 0.05) **O > R **(*negative fasting HDL cholesterol change*) (p < 0.05) **O > R **(*male negative fasting HDL cholesterol change (week 52 only)*) (p < 0.05) **R > O & Q **(*prolactin level change*) (p < 0.001) **Q > R **(*systolic blood pressure increase*) (p < 0.01) **O > R **(*systolic blood pressure increase*) (p < 0.05) **O > R **(*diastolic blood pressure increase (week 52 only)*) (p < 0.05) **O > R **(*increase of proportion with systolic blood pressure ≥ 130 mm Hg (week 52 only)*) (p < 0.05) **Q > R **(*increase of proportion with diastolic blood pressure ≥ 85 mm Hg (week 52 only*)) (p < 0.05)
Robinson et al. [26]	**O = R **(*SAS, BARS*) **O > R **(*weight gain*) (p < 0.01)
	
**ACUTE PHASE**	
	
Chrzanowski et al. [27]	**A = O **(*insomnia, anxiety, headache, somnolence, infection, nervousness, akathisia, schizophrenic reaction, flu syndrome, CNS stimulation, lightheadedness, tremor, SAS, BARS, AIMS, triglyceride change*) **O > A **(*Weight gain (p < 0.001, Weight gain > 7% (p = 0.008), elevated total (p < 0.01) and LDL cholesterol (p < 0.01), negative influence on HDL cholesterol (p < 0.05), QTc prolongation (p = 0.008), prolactin elevation *(p < 0.001)
Kraus et al. [28]	No outcomes
McCue et al. [29]	**A = H = O = Q = R = Z **(*SAS, BARS, spontaneous reports of adverse events*) **A = H = O = Q = R = Z **(*withdrawals because of side-effects*)
	
**CHRONIC PHASE**	
	
Jerrel [30]	**O = R = FGA **(*DISCUS, SAS, BARS*)
Lieberman et al. [31]	**O = P = Q = R = Z **(*any moderate or severe adverse effect, suicide attempt or ideation, hypersomnia, sleepiness, decreased sex drive, arousal, ability to reach orgasm, gynecomastia, galactorrhoea, menstrual irregularities, orthostatic faintness, AIMS, BARS, SAS, discontinuation owing to sedation, change from baseline of blood glucose, change in corrected QT interval, new cataracts*) **O > Q > R > P > Z **(*weight gain > 7%, mean cholesterol and -triglyceride change*) (p < 0.001); (*meanHbA1c change*) (p = 0.01) O > Q > R > Z* > P* (*weight change*) (p < 0.001) **O > Z > P > R > Q **(*HbA1c change*) (p = 0.01) O > Q > P > R* > Z* (*cholesterol change, triglyceride change*) (p < 0.001) **O > Q > Z > R > P **(*discontinuation owing to weight gain or metabolic effects*) (p < 0.001) **O > Q = P = Z > R **(*discontinuation owing to intolerability*) (p = 0.04) **Q > R > O > P > Z **(*urinary hesitancy, dry mouth, constipation*) (p < 0.001) **Z > P > R > Q > O **(*insomnia*) (p < 0.001) **R > O = Z > Q > P **(*incontinence, nocturi*) (p = 0.04) **P > Z > Q = R > O **(*discontinuation owing to EPS*) (p = 0.002) **R > P > >Z > O > Q **(*mean prolactin change*)(p < 0.001) **Q & R > O & Z **(*prolonged QTc interval*) (p < 0.03)
Mullen et al. [32]	**Q = R **(*EPS total (EPS checklist), odds of having an EPS event*) **R > Q **(*for odds of EPS of at least moderate severity *(p = 0.03), *odds of substantial EPS *(p < 0.001), *requirement of anti- EPS medication during the trial among baseline EPS patients *(p < 0.001) **Q > R **(*somnolence, dry mouth, dizziness, agitation*) (p < 0.05)
Ritchie et al. [33]	**O = R **(*SAS, AIMS, BARS, specific side effects*)
Ritchie et al. [34]	**O = R **(*SAS, AIMS, BARS, sedation, hypotension, dizziness, gastrointestinal side effects, libido, anticholinergics symptoms, weight gain*)
Rosenheck et al. [35]	No outcomes
Stroup et al. [36]	**O = Q = R = Z **(*hypersomnia, sleepiness, urinary hesitancy/dry mouth/constipation, incontinence/nocturia, AIMS, BARS, SAS, discontinuation because of intolerable extrapyramidal side effects or sedation, weight change over course of treatment, change of blood glucose and HbA1c, change in QTc interval*) **O > Q = R > Z **(> 7% *weight gain*) (p = 0.009) O > Q > R* > Z * (*average change in weigh and cholesterol change*) (p < 0.001) O > Q > Z* > R* (*mean change in triglycerides*) (p < 0.001) **Q > O > R > Z **(*orthostatic faintness*) (p < 0.05);*discontinuation because of intolerable weight or metabolic side effects*) (p = 0.004) **Q > R > Z > O ***(skin rash*) (p < 0.05) **Q > Z > O > R **(*any spontaneous report of moderate/severe adverse effect*) (p < 0.02)) **R > O > Z > Q **(*adverse events related to sex drive/sexual arousal/sexual orgasm*) (p < 0.05) **R > O = Z > Q **(*gynecomastia/galactorrhoea*) (p < 0.04) R > Z* > O* > Q* (*change of prolactin level*) (p < 0.001) **Z > R > Q > O **(*any serious adverse event*) (p = 0.01) **Z > R >Q > O **(*insomnia*) (p < 0.001)
Stroup et al. [37]	**O = Q = R **(*any moderate, severe or serious adverse event, insomnia, hypersomnia, sleepiness, urinary hesitancy, dry mouth, constipation, decreased sexual drive, sexual arousal, orgasm, incontinence, nocturia, sialorrhoea, orthostatic faintness, skin rash, AIMS, weight gain > 7%, rate of weight gain, change of blood glucose, HbA1c, QTc interval*) **O > Q > R **(*amount of weight gain*) (p = 0.005;, (*triglyceride change*) (p = 0.03) **O > R > Q **(*total cholesterol change*) (p = 0.01) R > Q* > O* (*change of prolactin level*) (p < 0.001)
Swartz et al. [38]	No outcomes
Tunis et al. [39]	**O = R = FGAS **(any *serious AE*) **O > FGA **(*probability of not developing EPS over 1 year among those without EPS at baseline*) (p = 0.006) **R **(p = 0.023) **& FGAs **(p < 0.0001) > **O **(*time to 7% weight gain*) **O > R > FGA **(*weight gain for patients on initial antipsychotic regimen) *(significant^1^, p = ?)

**Abbreviations: **A = Aripiprazole, FGAs = First generation antipsychotics, O = Olanzapine, P = Perphenazine, Q = Quetiapine, R = Risperidone, Z = Ziprasidone, DISCUS = the Dyskinesia Identification System Condensed User Scale, BARS = Barnes Akathisia Scale, AIMS = the Abnormal Involuntary Movement Scale, BARS = the Barnes Akathisia Rating Scale, SAS = the Simpson Angus Scale, UKU = UKU Side Effect Rating Scale, EPS = the extrapyramidal syndrome, BMI = Body Mass Index.

* = negative change

^1 ^The authors state that the difference is significant, but do not reveal the p-value for this comparison.

[24], [25] and [27] provide analyses on both last observation carried forward (LOCF) and observed cases. The main findings are the same, but only p-values for LOCF data are displayed in the table.

### Concomitant medication

On the use of concomitant medications (Table 5) there were no consistent differences between the SGAs.

**Table 5:** Concomitant medication

**Studies**	**Comparators**
**FIRST EPISODE**	
	
Crespo-Facorro et al. [24]	**H = O = R **(*benzodiazepines for anxiety/agitation or EPS; hypnotics*) **H > R > O **(*anticholinergics for EPS*) (p < 0.0001)
McEvoy et al. [25]	**O = Q = R **(*postbaseline adjunctive medication use for dysphoria/depression, anxiety, insomnia, agitation/excitement*) **O > Q **(*proportion receiving concomitant medications for parkinsonism or akathisia*) (p = 0.021)
Robinson et al. [26]	**O = R **(*divalproex, sertraline, benztropin, propranolol, lorazepam*)
	
**ACUTE PHASE**	
	
Chrzanowski et al. [27]	**A = O **(*anticholinergics*)
Kraus et al.* [28]	**O = R **(*benztropine and/or trihexyphenidyl, mood-stabilizers, antidepressants, haloperidol, quetiapine, ziprasidone, perphenazine, lorazepam and/or clonazepam*) **R > O **(*diphenhydramine and/or hydroxyzine*) (p = 0.05)
McCue et al. * [29]	**A = H = O = Q = R = Z **(*dosages of additional: haloperidol, lorazepam, benzatropine*) **A = H = O = Q = R = Z **(*patients receiving additional: mood stabiliser, antidepressant, anxiolytic*) **A > Q > R > Z > H > O **(*dosage of diphenhydramine*) (p = 0.004) **H > R > Q = Z > A = O **(*patients receiving anticholinergics*) (p < 0.0001)
	
**CHRONIC PHASE**	
	
Jerrel * [30]	**O & R > FGA **(*odds of being prescribed mood-stabilizers or supplemental antipsychotics*) (p < 0.001)
Lieberman et al. [31]	**O = P = Q = R = Z **(*lithium, anticonvulsants, oral glucose-lowering drugs, insulin, cholestatin drugs*) **R > Z > O > P > Q **(*antidepressants*) (p = 0.03) **R = P = Z > O > Q **(*hypnotics, sedatives*) (p = 0.03) **P = Z > Q > R > O **(*anxiolytics*) (p < 0.001) **P > R > Z > O > Q **(*anticholinergics agents*) (p = 0.01)
Mullen et al.* [32]	Not disclosed
Ritchie et al. [33]	Not disclosed
Ritchie et al. [34]	Not disclosed
Rosenheck et al. [35]	Not disclosed
Stroup et al. [36]	**O = Q = R = Z **(*details not disclosed*)
Stroup et al. [37]	**O = Q = R **(*lithium, anticonvulsants, antidepressants, hypnotic, sedatives, anxiolytics, anticholinergics, oral glucose-lowering drugs and insulin, cholestatin drugs*)
Swartz et al. [38]	Not disclosed
Tunis et al. [39]	Details not disclosed

**Abbreviations: **A = Aripiprazole, FGAs = First generation antipsychotics, O = Olanzapine, P = Perphenazine, Q = Quetiapine, R = Risperidone, Z = Ziprasidone, EPS = the extrapyramidal syndrome, * = Additional antipsychotics permitted.

### Funding

Six studies were supported by pharmaceutical companies. Reported findings of differences between comparator SGAs were in favour of the supporter's drugs in 5 of these studies [27,32-34,39], whereas one study found equal effectiveness among the comparators [25].

## Discussion

In this literature search 16 reports were located with comparisons of SGAs in clinical settings, and performed with the basic methodological demands such as randomization fulfilled. With regards to global outcomes the most consistent finding was a superior drug adherence or time to treatment discontinuation (drug survival) for olanzapine in patients suffering from chronic schizophrenia. Drug adherence and survival were considered global effectiveness outcome measures, as they were thought to reflect both efficacy and tolerability of the drugs as judged by both the patient and treating psychiatrist. The outcome measure is clinically important, as antipsychotic drug adherence has major influences on risks of relapse, rehospitalisation and suicide in patients with schizophrenia [40]. Three of the five studies using this outcome measure were from the CATIE trial. A critical question is whether the comparator drugs were used in equivalent doses in the CATIE studies [41]. To permit blinding combined with flexible-dose regimens in the CATIE study, drug doses representing a quartile of the maximal daily drug dose were packaged in 4 capsules that were identical-appearing for all study drugs. The matter of choice of upper dose limit has substantial impact upon the individual steps in the up-titration of the drugs. The upper dose limit for olanzapine is, in contrast to the other drugs, set above the label-defined upper dosage limit of 20 mg [31]. This causes bigger up-titration steps for olanzapine than the comparators, as each up-titration step correlates with more "response" for olanzapine compared to the other drugs under investigation. The fact that the studies by Jerrel [30] and Tunis et al. [39] have similar results to the ones from the CATIE despite the use of lower doses of olanzapine supports the finding of superiority of olanzapine on treatment adherence, however.

Regarding ability to alleviate symptoms of psychosis, the drugs performed equally in all acute phase studies and all but one chronic phase studies. The solitary CATIE study that found olanzapine to be superior to quetiapine and ziprasidone had a sample of chronic schizophrenia patients that had previously discontinued an SGA because of intolerability. In this study the mean DDD of olanzapine was 2.05 compared to 1.41 and 1.45 for quetiapine and ziprasidone respectively. The difference in total PANSS response may in line with the above mentioned argument be a result of non-equivalent doses. In the study by Tunis et al. [39] olanzapine had more clinical responder days compared to risperidone, as defined by mean number of days with scores of the Brief Psychiatric Rating Scale (BPRS) less than 18. This finding does not necessarily imply that the olanzapine group was more effective than the risperidone group as measured by total reduction of BPRS. The latter comparison is not disclosed in the paper.

The tolerability outcomes were somewhat surprising as the SGAs performed equally on most measures. Maybe the most striking finding was the lack of differences between the SGAs with regard to the extrapyramidal syndrome and related side effects across all studies. This might indicate that the drugs' distinct side effect profiles derived from efficacy trials are "levelled out" at least to some degree in the naturalistic setting where samples are more heterogeneous and concomitant psychotropics are less restricted. It is worth noting that some of the studies have rather low sample sizes, which increases the risk of statistical type 2 errors and thereby failure to detect real differences between drugs. The most pronounced difference between the SGAs was in the area of metabolic adverse effects, where olanzapine-treated patients gained more weight and had the most adverse influence on cholesterol and triglycerides levels.

Six studies included a FGA arm in the design and two of these studies found the FGA(s) to be associated with lower total mental health care costs [30,35], whereas one study found equal total costs between olanzapine, risperidone and the FGAs haloperidol and perphenazine [39]. The latter study was supported by industry. Cost-effectiveness measurements were not included in the rest of the studies. Based on the present review the SGAs were not superior to FGAs with regards to treating symptoms of disease. The FGAs were associated with more EPS and related adverse effects in 3 of the studies involving a FGA arm, however. Important factors not reviewed in the present paper include the potential differential effects of SGAs versus FGAs on cognitive impairments and risk of relapse. There are some indications that SGAs are superior to FGAs on these outcome measures [42,43]. Before these issues are further investigated in effectiveness studies it would be premature to properly estimate cost-benefit of the drugs.

**Figure 1:**
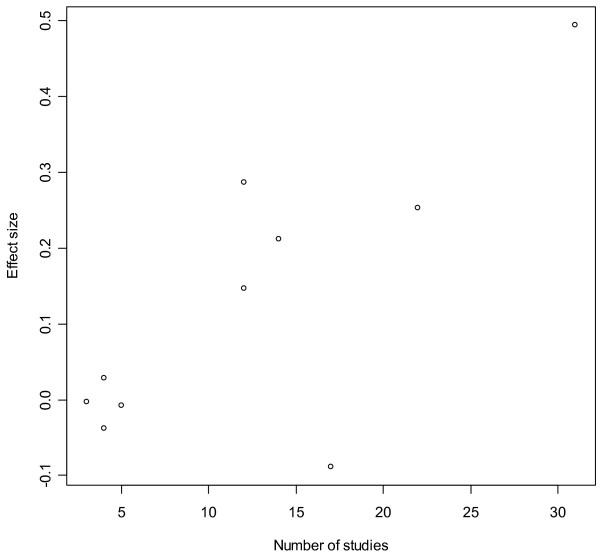
**Effect sizes of second generation antipsychotics compared with first generation antipsychotics**. Relationship between number of studies on the individual drugs, and effect sizes of the respective 10 second generation antipsychotics compared to first generation antipsychotics. Adapted from Table 2 in Davis et al. [44]

One third of the studies were funded by the pharmaceutical industry and in these studies main findings of differences between the SGAs were in favour of the funder's product in 5 of 6 cases. In recent years the matter of conflict of interest in research has become a major concern as a high number of psychotropic drug trials are financially supported by the industry, and "funding bias" has been pin-pointed by several authors [44-49]. In a review of head-to-head RCT comparisons of SGAs, outcomes were in favour of the funding party in 9 out of 10 studies, which also led to contradictory results in studies from different sources of sponsorship [50]. In a recent meta-analysis by Davis et al. [51] on the efficacy of SGAs, the conclusion is that some of the SGAs are more efficacious than others when compared to FGAs. Davis et al. state that almost all the studies in their analysis were supported by industry. In the SGA vs. FGA comparisons it is reasonable to presume without exception that the FGAs are the reference drugs and that sponsorships are strongly associated with the SGAs. Interestingly, the SGAs with the highest effect sizes were also the ones represented with the largest number of studies (Figure 1). In fact, there seems to be an almost linear relationship between number of studies on, and effect sizes for individual SGAs, with remoxipride being the only deviator. If there is a systematic bias that favours the drug of the funding party, this could at least in part explain the obvious "dose-response" relationships observed in Figure 1, with number of studies being the "dose" and effect size being the "response". Results from both efficacy and effectiveness studies funded by the pharmaceutical industry should accordingly be interpreted bearing the possibility of "funding bias" in mind.

The present sixteen studies were all performed according to naturalistic designs. However, certain methodological differences reflect that the concepts of effectiveness trials and of naturalistic/real life/pragmatic/practical approaches are not strictly defined. There are obvious differences between the studies' samples (Table 1). This fact makes it very dubious to pool the rates from individual studies in meta-analyses and for instance calculate joint effect-sizes ("the apples and oranges error) [52]. To bypass this source of bias, we presented the main results from each study separately and with emphasis on statistically significant differences between the SGAs excluding absolute figures or rates. This of course represents a crude method but is well suited in search of robust differences between the SGAs. Four studies permitted the use of additional antipsychotics which could confound comparisons between the SGAs [28-30,32]. No differences were found between the SGAs in the use of supplemental antipsychotics, however. Another limitation of this review is that only 3 studies included first-episode patients of which 1 study was of very short duration. From a clinical point of view, the first-time antipsychotic intervention is the one associated with the highest degree of uncertainty. In the chronic patient, prior experience with antipsychotics may deliver valuable information in the decision making about choosing an antipsychotic drug. For the drug-naïve, physicians are forced to perform sometimes extensive drug "trials" in the individual patient before an antipsychotic drug with satisfying effect profile is identified. Besides the economical aspects and the strain on the patient, this "trial" approach that may take several months extends the duration of untreated psychosis which may be a negative prognostic factor. More effectiveness studies on first-episode patients and with longer follow-up are called for.

## Conclusion

Despite the limitations mentioned above we conclude that in chronically ill patients olanzapine may have an advantage over other SGAs regarding longer time to treatment discontinuation and a better drug adherence, but olanzapine is also associated with more metabolic side effects. The SGAs were equally associated with EPS and related side effects.

More studies on first-episode psychosis are needed.

## Competing interests

The authors declare that they have no competing interests.

## Authors' contributions

EJ did the literature search and drafted the manuscript. HAJ participated in the design of the review and helped to draft the manuscript. Both authors read and approved the final manuscript.

## Pre-publication history

The pre-publication history for this paper can be accessed here:



## References

[B1] Arlington VA, American Psychiatric Association (2004). Practice guidelines for the treatment of patients with schizophrenia.

[B2] Marder SR, Essock SM, Miller AL, Buchanan RW, Davis JM, Kane JM, Lieberman J, Schooler NR (2002). The Mount Sinai conference on the pharmacotherapy of schizophrenia.. Schiz Bull.

[B3] Moore TA, Buchanan RW, Buckley PF, Chiles JA, Conley RR, Crismon ML, Essock SM, Finnerty M, Marder SR, Miller del D, McEvoy JP, Robinson DG, Schooler NR, Shon SP, Stroup TS, Miller AL (2007). The Texas medication algorithm project antipsychotic algorithm for schizophrenia: 2006 update. J Clin Psychiatry.

[B4] National Institute for Clinical Excellence (2002). Guidance on the use of newer (atypical) antipsychotic drugs for the treatment of schizophrenia. Technology appraisal guidance – No43.

[B5] Bagnall A, Kleijnen J, Leitner M, Lewis R (2000). Ziprasidone for schizophrenia and severe mental illness. Cochrane Database of Systematic Reviews.

[B6] Duggan L, Fenton M, Rathbone J, Dardennes R, El-Dosoky A, Indran S (2005). Olanzapine for schizophrenia. Cochrane Database of Systematic Reviews.

[B7] El-Sayeh HG, Morganti C (2006). Aripiprazole for schizophrenia. Cochrane Database of Systematic Reviews.

[B8] Gilbody SM, Bagnall AM, Duggan L, Tuunainen A (2000). Risperidone versus other atypical antipsychotic medication for schizophrenia. Cochrane Database of Systematic Reviews.

[B9] DeSilva P, Fenton M, Rathbone J (2006). Zotepine for schizophrenia. Cochrane Database of Systematic Reviews.

[B10] Rummel C, Hamann J, Kissling W, Leucht S (2003). New generation antipsychotics for first episode schizophrenia. Cochrane Database of Systematic Reviews.

[B11] Srisurapanont M, Maneeton B, Maneeton N (2004). Quetiapine for schizophrenia. Cochrane Database of Systematic Reviews.

[B12] Tuunainen A, Wahlbeck K, Gilbody SM (2000). Newer atypical antipsychotic medication versus clozapine for schizophrenia. Cochrane Database of Systematic Reviews.

[B13] Rosenheck R, Perlick D, Bingham S, Liu-Mares W, Collins J, Warren S, Leslie D, Allan E, Campbell EC, Caroff S, Corwin J, Davis L, Douyon R, Dunn L, Evans D, Freeska E, Grabowski J, Graaeber D, Herz L, Kwon K, Lawson W, Mena F, Sheikh J, Smelson D, Smith-Gamble V (2003). Effectiveness and cost of Olanzapine and haloperidol in the treatment of schizophrenia. JAMA.

[B14] Bowen J, Hirsch S (1992). Recruitment rates and factors affecting recruitments for a clinical trial of a putative anti-psychotic agent in the treatment of acute schizophrenia. Hum Psychopharmacol.

[B15] Robinson D, Woerner M, Pollack S, Lerner G (1996). Subject selection biases in clinical trials: Data from a multicenter schizophrenia treatment study. J Clin Psychopharmacol.

[B16] Hofer A, Hummer M, Huber R, Kurz M, Walch T, Fleischhacker WW (2000). Selection bias in clinical trials with antipsychotics. J Clin Psychopharmacol.

[B17] Johnsen E, Svingen GF, Jørgensen HA (2004). Practice regarding antipsychotic therapy: A cross-sectional survey in two Norwegian hospitals. Nord J Psychiatry.

[B18] Martin K, Bègaud B, Verdoux H, Lechevallier N, Latry P, Moore N (2004). Patterns of risperidone prescription: a utlilization study in south-west France. Acta Psychiatr Scand.

[B19] Ganguli R, Kotzan JA, Miller LS, Kennedy K, Martin DM (2004). Prevalence, trends, and factors associated with antipsychotic polypharmacy among Medicaid-eligible schizophrenia patients, 1998–2000. J Clin Psychiatry.

[B20] Edlinger M, Hausmann A, Kemmler G, Kurz M, Kurzhaler I, Walch T, Walpoth M, Fleischhacker WW (2005). Trends in the pharmacological treatment of patients with schizophrenia over a 12 year observation period. Schizophr Res.

[B21] Jaffe AB, Levine J (2003). Efficacy and effectiveness of first- and second-generation antipsychotics in schizophrenia. J Clin Psychiatry.

[B22] Nasrallah HA, Targum SD, Tandon R, McCombs JS, Ross R (2005). Defining and measuring clinical effectiveness in the treatment of schizophrenia. Psychiat Serv.

[B23] Arlington VA, American Psychiatric Association (2000). Practice guideling for the treatment of patients with major depressive disorder.

[B24] Crespo-Facorro B, Pérez-Iglesias R, Ramirez-Bonilla M, Martinéz-García O, LLorca J, Vásquez-Barquero JL (2006). A practical clinical trial comparing haloperidol, risperidone, and Olanzapine for the acute treatment of first-episode nonaffective psychosis. J Clin Psychiatry.

[B25] McEvoy JP, Lieberman JA, Perkins DO, Hamer RM, Gu H, Lazarus A, Sweitzer D, Olexy C, Weiden P, Strakowsky SD (2007). Efficacy and tolerability of olanzapine, quetiapine, and risperidone in the treatment of early psychosis: a randomized, double-blind 52-week comparison. Am J Psychiatry.

[B26] Robinson DG, Woerner MG, Napolitano B, Patel RC, Sevy SM, Gunduz-Bruce H, Soto-Perello JM, Mendelowitz A, Khadivi A, Miller R, McCormack J, Lorell BS, Lesser ML, Schooler NR, Kane JM (2006). Randomized comparison of Olanzapine versus risperidone for the treatment of first-episode schizophrenia: 4-month outcomes. Am J Psychiatry.

[B27] Chrzanowski WK, Marcus RN, Torbeyns A, Nyilas M, McQuade RD (2006). Effectiveness of long-term aripiprazole therapy in patients with acutely relapsing or chronic, stable schizophrenia: a 52-week, open-label comparison with olanzapine. Psychopharmacology.

[B28] Kraus JE, Sheitman BB, Cook A, Reviere R, Lieberman JA (2005). Olanzapine versus risperidone in newly admitted acutely ill psychotic patients. J Clin Psychiatry.

[B29] McCue RE, Waheed R, Urcuyo L, Orendain G, Joseph MD, Charles R, Hasan SM (2006). Comparative effectiveness of second-generation antipsychotics and haloperidol in acute schizophrenia. Br J Psychiatry.

[B30] Jerrel JM (2002). Cost-effectiveness of risperidone, Olanzapine, and conventional antipsychotic medications. Schizophr Bull.

[B31] Lieberman JA, Stroup TS, McEvoy JP, Swartz MS, Rosenheck RA, Perkins DO, Keefe RSE, Davis SM, Davis CE, Lebowitz BD, Severe J, Hsiao JK (2005). Effectiveness of antipsychotic drugs in patients with chronic schizophrenia. N Engl J Med.

[B32] Mullen J, Jibson MD, Sweitzer D (2001). A comparison of the relative safety, efficacy, and tolerability of quetiapine and risperidone in outpatients with schizophrenia and other psychotic disorders: The quetiapine experience with safety and tolerability (QUEST) study. Clin Ther.

[B33] Ritchie CW, Chiu E, Harrigan S, Hall K, Hassett A, Macfarlane S, Mastwyk M, O'Connor DW, Opie J, Ames D (2003). The impact upon extra-pyramidal side effects, clinical symptoms and quality of life of a switch from conventional to atypical antipsychotics (risperidone or olanzapine) in elderly patients with schizophrenia. Int J Geriatr Psychiatry.

[B34] Ritchie CW, Chiu E, Harrigan S, Macfarlane S, Mastwyk M, Halliday G, Hustig H, Hall K, Hassett A, O'Connor DW, Opie J, Nagalingam V, Snowdon J, Ames D (2006). A comparison of the efficacy and safety of olanzapine and risperidone in the treatment of elderly patients with schizophrenia: an open study of six months duration. Int J Geriatr Psychiatry.

[B35] Rosenheck RA, Leslie DL, Sindelar J, Miller EA, Lin H, Stroup TS, McEvoy J, Davis SM, Keefe RSE, Swartz M, Perkins DO, Hsiao JK, Lieberman J (2006). Cost-effectiveness of second-generation antipsychotics and perphenazine in a randomized trial of treatment for chronic schizophrenia. Am J Psychiatry.

[B36] Stroup TS, Lieberman JA, McEvoy JP, Swartz MS, Davis SM, Rosenheck RA, Perkins DO, Keefe RSE, Davis CE, Severe J, Hsiao JK (2006). Effectiveness of olanzapine, quetiapine, risperidone, and ziprasidone in patients with chronic schizophrenia following discontinuation of a previous atypical antipsychotic. Am J Psychiatry.

[B37] Stroup TS, Lieberman JA, McEvoy JP, Swartz MS, Davis SM, Capuano GA, Rosenheck RA, Keefe RSE, Miller AL, Belz JK (2007). Effectiveness of Olanzapine, quetiapine, and risperidone ion patients with chronic schizophrenia after discontinuing perphenazine: A CATIE study. Am J Psychiatry.

[B38] Swartz MS, Perkins KO, Stroup TS, Davis SM, Capuano G, Rosenheck RA, Reimherr F, McGee MF, Keefe RSE (2007). Effects of antipsychotic medications on psychosocial functioning in patients with chronic schizophrenia: Findings from the NIMH CATIE study. Am J Psychiatry.

[B39] Tunis SL, Faries DE, Nyhuis AW, Kinon BJ, Ascher-Svanum H, Aquila R (2006). Cost-effectiveness of olanzapine as first-line treatment for schizophrenia: Results from a randomized, open-label, 1-year trial. Value Health.

[B40] Leucht S, Heres S (2006). Epidemiology, clinical consequences, and psychosocial treatment of nonadherence in schizophrenia. J Clin Psychiatry.

[B41] Meltzer HY, Bobo WV (2006). Interpreting the efficacy findings in the CATIE study: What clinicians should know. CNS Spectr.

[B42] Keefe RSE, Silva SG, Perkins DO, Lieberman JA (1999). The effects of atypical antipsychotic drugs on neurocognitive impairment in schizophrenia: a review and meta-analysis. Schizophrenia Bull.

[B43] Leucht S, Barnes TRE, Kissling W, Engel RR, Correll C, Kane JM (2003). Relapse prevention in schizophrenia with new-generation antipsychotics. A systematic review and exploratory meta-analysis of randomized, controlled trials. Am J Psychiatry.

[B44] Perlis RH, Perlis CS, Wu Y, Hwang C, Joseph M, Nierenberg AA (2005). Industry sponsorship and financial conflict of interest in the reporting of clinical trials in psychiatry. Am J Psychiatry.

[B45] Lexchin J, Bero LA, Djubegovic B, Clark O (2003). Pharmaceutical industry sponsorship and research outcome and quality: systematic review. Br Med J.

[B46] Yaphe J, Edman R, Knishkowy B, Herman J (2001). The association between funding by commercial interests and study outcome in randomized controlled trials. Family practice.

[B47] Kjaergard LL, Als-Nielsen B (2002). Association between competing interests and authors' conclusions: epidemiological study of randomized clinical trials in the BMJ. Br Med J.

[B48] Clifford TJ, Barrowman NJ, Moher D (2002). Funding source, trial outcome and reporting quality: are they related. Results of a pilot study. BMC Health Serv Res.

[B49] Djulbegovic B, Lacevic M, Cantor A, Fields K, Bennett CL, Adams JR, Kuderer NM, Lyman GH (2000). The uncertainty principle and industry-sponsored research. Lancet.

[B50] Heres S, Davis J, Maino K, Jetzinger E, Kissling W, Leucht S (2006). Why Olanzapine beats risperidone, risperidone beats quetiapine, and quetiapine beats Olanzapine. An exploratory analysis of head-to-head comparisons studies of second-generation antipsychotics. Am J Psychiatry.

[B51] Davis JM, Chen N, Glick ID (2003). A meta-analysis of the efficacy of second-generation antipsychotics. Arch Gen Psychiatry.

[B52] Shirzadi AA, Ghaemi SN (2006). Side effects of atypical antipsychotics: extrapyramidal symptoms and the metabolic syndrome. Harv Rev Psychiatry.

